# Hypertensive Crisis-Related Hospitalizations and Subsequent Major Adverse Cardiac Events in Young Adults with Cannabis Use Disorder: A Nationwide Analysis

**DOI:** 10.3390/medicina58101465

**Published:** 2022-10-16

**Authors:** Rupak Desai, Akhil Jain, Waleed Sultan, Zainab Gandhi, Athul Raj Raju, Vivek Joseph Varughese, Geethu Jnaneswaran, Charu Agarwal, Bisharah Rizvi, Zeeshan Mansuri, Puneet Gupta, Gautam Kumar, Rajesh Sachdeva

**Affiliations:** 1 Division of Cardiology, Atlanta VA Medical Center, 1670 Clairmont Rd., Decatur, GA 30033, USA; 2 Department of Internal Medicine, Mercy Catholic Medical Center, Darby, PA 19153, USA; 3 Department of Family Medicine, Conemaugh Memorial Medical Center, Johnstown, PA 15905, USA; 4 Department of Internal Medicine, Geisinger Wyoming Valley Medical Center, Wilkes-Barre, PA 18711, USA; 5 Department of Medicine, Karuna Medical College, Chittur-Thathamangalam 678103, Kerala, India; 6 Department of Internal Medicine, Government Medical College, Thiruvananthapuram 695011, Kerala, India; 7 Department of Medicine, SUT Academy of Medical Sciences, Thiruvananthapuram 695028, Kerala, India; 8 Department of Medicine, Sri Siddhartha Medical College, Tumakuru 572107, Karnataka, India; 9 Department of Internal Medicine, Saint Agnes Medical Center, Fresno, CA 93720, USA; 10 Department of Psychiatry, Boston Children’s Hospital, Harvard Medical School, Boston, MA 02115, USA; 11 Department of Cardiology, Baptist Health Deaconess Madisonville, Madisonville, KY 42431, USA; 12 Division of Cardiology, Emory University School of Medicine, Atlanta, GA 30307, USA

**Keywords:** cannabis, marijuana, hypertension, hypertensive crisis, hypertensive emergency, in-hospital mortality, young

## Abstract

*Background and Objectives*: With the growing recreational cannabis use and recent reports linking it to hypertension, we sought to determine the risk of hypertensive crisis (HC) hospitalizations and major adverse cardiac and cerebrovascular events (MACCE) in young adults with cannabis use disorder (CUD+). *Material and Methods*: Young adult hospitalizations (18–44 years) with HC and CUD+ were identified from National Inpatient Sample (October 2015–December 2017). Primary outcomes included prevalence and odds of HC with CUD. Co-primary (in-hospital MACCE) and secondary outcomes (resource utilization) were compared between propensity-matched CUD+ and CUD- cohorts in HC admissions. *Results*: Young CUD+ had higher prevalence of HC (0.7%, *n* = 4675) than CUD- (0.5%, *n* = 92,755), with higher odds when adjusted for patient/hospital-characteristics, comorbidities, alcohol and tobacco use disorder, cocaine and stimulant use (aOR 1.15, 95%CI:1.06–1.24, *p* = 0.001). CUD+ had significantly increased adjusted odds of HC (for sociodemographic, hospital-level characteristics, comorbidities, tobacco use disorder, and alcohol abuse) (aOR 1.17, 95%CI:1.01–1.36, *p* = 0.034) among young with benign hypertension, but failed to reach significance when additionally adjusted for cocaine/stimulant use (aOR 1.12, *p* = 0.154). Propensity-matched CUD+ cohort (*n* = 4440, median age 36 years, 64.2% male, 64.4% blacks) showed higher rates of substance abuse, depression, psychosis, previous myocardial infarction, valvular heart disease, chronic pulmonary disease, pulmonary circulation disease, and liver disease. CUD+ had higher odds of all-cause mortality (aOR 5.74, 95%CI:2.55–12.91, *p* < 0.001), arrhythmia (aOR 1.73, 95%CI:1.38–2.17, *p* < 0.001) and stroke (aOR 1.46, 95%CI:1.02–2.10, *p* = 0.040). CUD+ cohort had fewer routine discharges with comparable in-hospital stay and cost. *Conclusions*: Young CUD+ cohort had higher rate and odds of HC admissions than CUD-, with prevalent disparities and higher subsequent risk of all-cause mortality, arrhythmia and stroke.

## 1. Introduction

Legalization and decriminalization of cannabis use may account for the unmonitored use of cannabis. Yu et al. found a link between cannabis usage among adolescents and young adults and laws and regulations in the United States since 1950 using an age-period-cohort model [1]. The physiological effects of cannabis usage, particularly in young individuals who are more prone to do it recreationally, are contradictory and lacking in evidence. Cannabis is being advocated for its therapeutic benefits in terms of its anti-inflammatory and analgesic actions, and studies have shown a few positive effects of cannabidiol in experimental models of heart diseases (myocardial infarction, cardiomyopathy, myocarditis, stroke, etc.) by decreasing organ damage, oxidative and nitrative stress, inflammatory processes, and apoptosis [2]. As much as the preliminary reports of the potential benefits of the medicinal use of cannabis are encouraging, previous studies revealing detrimental effects of chronic or habitual recreational cannabis use raise concern. In our cross-sectional study using the National Inpatient Sample between 2007 and 2014, we found alarmingly rising trends in hypertensive emergency-related admissions in cannabis users [3]. A recent study also reported nearly one-third of the study subjects, predominantly young males, experienced tachycardia and hypertension with the use of synthetic cannabinoids [4]. Synthetic cannabinoids have a 100–200 times greater effect on Cannabinoid receptor type 1 than tetrahydrocannabinol and have been classed as a prohibited substance due to its negative effects [5]. Low doses of cannabinoids have been linked to an enhanced sympathetic response (tachycardia, hypertension, and contractility), as well as elevated norepinephrine levels measured 30 min after usage [6]. The available research on the effect of cannabis on blood pressure, on the other hand, appears to be contradictory. A large-population observational study showed no association of hypertension with cannabis use over a follow-up period of 12 months [7]. We evaluated the prevalence, causes, and effects of hypertensive crisis (HC) among young adults with cannabis use disorder (CUD+) to non-users in a retrospective analysis of a nationally representative cohort from the United States (US) (CUD-).

## 2. Methods

The National Inpatient Sample (NIS) is the largest publicly accessible all-payer inpatient database in the US as a part of the Healthcare Cost and Utilization Project (HCUP) [8]. The NIS data from October 2015 through December 2017 were used for this study as ICD-10 diagnosis and procedural codes were implemented throughout the US effective 1 October 2015. Young patients (aged 18 to 44 years) with CUD+ and hypertensive crisis were identified using International Classification of Diseases, Ninth and Tenth Revision, Clinical Modification (ICD-10-CM) diagnostic codes F12.1x and F12.2x (excluding F12.21 dependence in remission) and I16.x codes, respectively. The study population was divided into two groups: CUD+ vs. CUD- cohorts to assess the prevalence of HC hospitalizations, associated comorbidities and in-hospital outcomes. Owing to the deidentified nature of the NIS dataset, approval from the institutional review board was not mandatory.

The primary outcomes were prevalence and odds of HC-related admissions with demographic characterization and rate and predictors of subsequent in-hospital outcomes (mortality, other cardiovascular complications defined as major adverse cardiac and cerebrovascular events) in cannabis users. The secondary outcomes were healthcare resource utilization for HC hospitalizations in the CUD+ cohort, disposition patterns (routine, short-term hospital transfer, skilled or intermediate nursing facility, and other transfers), length of stay (LOS), and adjusted hospitalization costs per 2017 inflation data.

A two-tailed *p* < 0.05 was considered a threshold for clinical significance. Due to a substantial difference in the total number of valid observations between the two groups of all admissions with HC, a propensity-matched analysis was performed with a ratio of 1:1 without replacement using a caliper width of 0.01. The absolute standardized difference of <10% was obtained for most variables before and after propensity matching.

Data was matched with all baseline characteristics, comorbidities, and hospital characteristics. Only 1:1 propensity-matched data were utilized to assess primary and secondary outcomes. Chi-square test (categorical data reported in percentage) and Mann–Whitney U test (reporting median and interquartile range) were performed to compare the baseline characteristics. Outcomes and predictors were adjusted for age, sex, race, median income, payer status, hospital characteristics, and relevant comorbidities. Odds ratios (OR) and 95% confidence intervals (CI) were calculated for mortality predictors. IBM Statistical Package for the Social Sciences (SPSS) v24.0 (IBM Corp., Armonk, NY, USA) was utilized to perform the analyses.

## 3. Results

Out of 19,448,302 total hospitalizations among young adults (18–44 years) between October 2015 to December 2017, there were 623,715 [3.2%, median age 29 (24–36) years, 61.2% male] admissions in CUD+ arm and 18,824,587 [median 31 (26–37) years, 74.5% female] CUD- arm (Table 1). The CUD+ arm often had non-elective admissions compared to the CUD- arm (88% vs. 68.1%) and often consisted of African Americans (29.0% vs. 18.8%) and patients from lower-income quartiles (40.8 vs. 31.8%), *p* < 0.001) vs. the CUD- arm. The CUD+ cohort had more Medicaid enrollees (49.9 vs. 39.2%), whereas private insurances were the primary payers for the CUD- cohort (43.4 vs. 22.4%). The CUD+ arm demonstrated higher frequency of admissions in Northeastern and Midwestern hospitals compared to CUD- arm.

The comorbidities’ prevalence was assessed in both groups. AIDS, chronic pulmonary disease, liver disease, neurological disorders, depression and psychosis were found to be significantly more prevalent in the HC−CUD+ arm, whereas hypertension, diabetes, hyperlipidemia, obesity, peripheral vascular disease (PVD), rheumatoid/collagen vascular disease, coagulopathy, congestive heart failure, pulmonary circulation disease, renal failure, tumors with or without metastasis, and lymphoma were significantly more prevalent in HC+CUD- arm. The CUD+ arm had a higher rate of concomitant use of other addictive substances than CUD- arm-smoking (58.8 vs. 23.1%), alcohol abuse (19.1 vs. 3.9%) and overall drug abuse (86.2 vs. 5.5%) (*p* < 0.001).

**Figure 1:**
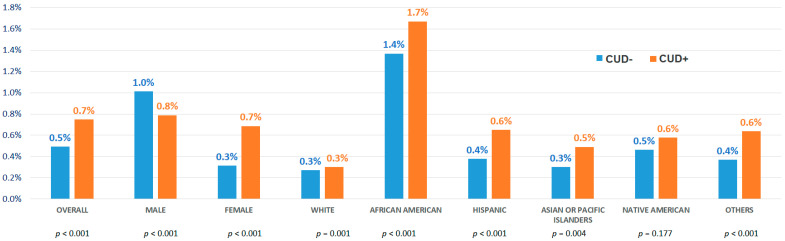
Hypertensive Crisis-related Hospitalizations in Young Adults (18–44 years) With vs. Without Cannabis Use Disorder.

The crude prevalence of HC was found to be higher in CUD+ cohort vs. CUD- cohort [*n* = 4675 (0.7%) vs. *n* = 92755 (0.5%), *p* < 0.05] (Figure 1). On the subgroup analyses, female (0.7% vs. 0.3%), African American (1.7% vs. 1.4%), Hispanic (0.6% vs. 0.4%) and Asian or Pacific Islander (API, 0.5% vs. 0.3%) patients with CUD+ demonstrated the higher crude prevalence of HC-related hospitalizations compared to CUD-.

As shown in Table 2, the unadjusted risk of HC admissions was higher in the overall young adult population with CUD; OR: 1.52 (95%CI: 1.41–1.64, *p* < 0.001) and also in young adults with known benign hypertension; OR: 1.25 (95%CI: 1.09–1.44, *p* = 0.002). Along with sociodemographic, hospital-level confounders, and pre-existing comorbidities, the multivariable analyses revealed significantly higher odds of HC; OR: 1.22 (95%CI:1.13–1.32, *p* < 0.001) when adjusted for alcohol abuse and tobacco use disorder, which remained high when the models were additionally adjusted for cocaine abuse and stimulant use including amphetamine OR:1.15 (95%CI: 1.06–1.24, *p* = 0.001).

Among young adults with known benign hypertension, CUD increased the odds of HC-related hospitalizations. The odds ratios were as follows- Unadjusted OR: 1.25 (95%CI: 1.09–1.44, *p* = 0.002), adjusted analysis for sociodemographic/hospital-level characteristics, comorbidities, tobacco use disorder, and alcohol abuse OR: 1.17 (95%CI: 1.01–1.36, *p* = 0.034), adjusted analysis for sociodemographic/hospital-level characteristics, comorbidities, tobacco use disorder, alcohol abuse, cocaine abuse and stimulant use including amphetamine OR: 1.12, (95%CI: 0.96–1.30, *p* = 0.154).

Propensity score-matched (1:1) cohorts (*n* = 4440 CUD+ vs. *n* = 4440 CUD-) showed a balanced distribution of most of the sociodemographic variables between the 2 arms (Table 3). Matched cohorts of HC admissions for CUD+ and non-CUD had a mean age of 36 and 37 years (*p* = 0.004). Matching confirmed a higher but non-significant trend for male admissions within the CUD+ cohort itself and also when compared to CUD- (*p* = 0.186) arm. More whites and API were admitted in the CUD+HC+ arm than the CUD- arm (20.8 vs. 18.9%), whereas blacks (65.1 vs. 64.4%), Hispanics (11.8 vs. 10.2%) and Native Americans (1.1 vs. 0.9%) had more admissions in the HC+CUD- arm. Differences were statistically significant for racial distribution with *p* = 0.029. Statistically non-significant differences were observed for the type of admission, type of admitting hospital, region of hospitalization, socioeconomic status, and primary payer on discharge. Matching confirmed the higher prevalence of smoking, alcohol abuse, and drug abuse in the CUD+ HC+ arm. Significant differences were found (*p* < 0.001) for tobacco (65.4 vs. 40.8%), alcohol (12.8 vs. 5.6%) and drug abuse (87.7 vs. 8.4%) between CUD+ and CUD- groups. Chronic pulmonary, liver disease, depression and psychosis were also significantly higher in the CUD+ HC+ cohort. Prior myocardial infarctions were more common in the CUD+ arm than CUD- (4.3 vs. 3.3%, *p* = 0.012). Comorbidities traditionally associated with increased cardiovascular disease burden—Diabetes mellitus, peripheral vascular disease, hyperlipidemia, obesity and renal failure were higher in the CUD- cohort than the CUD+ cohort (all *p* < 0.05). Congestive heart failure prevalence did not have a statistically significant difference between the two groups.

On a multivariable analysis adjusted for cardiovascular and extracardiac comorbidities along with socio-demographic and hospital characteristics, there were significantly higher odds of all-cause mortality (aOR 5.74, 95%CI 2.55–12.91, *p* < 0.001), arrhythmia (aOR 1.73, 95 CI 1.38–2.17, *p* < 0.001) and stroke (aOR 1.46, 95 CI 1.02–2.10, *p* = 0.04) in the CUD+ arm compared to the CUD- arm of all HC admissions in young adults. Acute myocardial infarction (AMI) and cardiac arrests had higher odds but did not reach statistical significance (Table 4). Furthermore, advancing age, admissions to Midwestern or Southern hospitals (compared to Northeastern hospitals) and comorbidities including AIDS, PVD, coagulopathy, prior history of TIA/stroke, and other neurological disorders independently increased the odds of MACCE in young adults with CUD admitted for HC (Table 5).

## 4. Discussion

To our knowledge, this is the largest population-based analysis to date reporting the burden and impact of CUD+ on HC and associated in-hospital outcomes in young adults using the nationwide cohorts in the US. Among the total hospitalizations in this age group, 3.2% were CUD-related admissions. This study revealed higher odds of HC in overall young population (aOR 1.22) and young adults with known benign hypertension (aOR 1.17) when adjusted for comorbid conditions, tobacco use disorder and alcohol abuse. Furthermore, the higher risk still persisted in overall young adult hospitalizations even after additional adjustment with cocaine abuse and stimulant use but young adults with known benign hypertension showed non-significantly higher odds of HC.

Unmatched CUD+ cohort often consisted of males (61.2% vs. 25.5%), blacks (29.0% vs. 18.8%), Medicaid enrollees (49.9% vs. 39.2%) and patients from the lowermost income quartile (40.8% vs. 31.8%) consistent to recent data from the 2015 National Survey that showed that individuals in poverty were three times more likely to suffer from CUD after controlling for gender, age, tobacco, and alcohol use [9]. Tobacco use, alcohol, drug abuse, depression, psychosis, history of previous myocardial infarction, valvular heart disease, chronic pulmonary disease, pulmonary circulation disease, and liver disease were more prevalent in CUD+ cohort than CUD- cohort.

The crude prevalence of the HC was higher in the CUD+ cohort as in comparison with the CUD- cohort. Similarly, when adjusted for demographics, hospital characteristics, and comorbid conditions, our analysis revealed higher odds of HC admission with CUD+. Available evidence on the effects of cannabis on blood pressure appears to be conflicting. Courts et al. reported that synthetic cannabinoid toxicity in young males is connected to cardiovascular symptoms, including tachycardia and hypertension [4]. In a study using a cross-sectional national survey, Vidot et al. reported that cannabis users had a higher prevalence of hypertension than non-users, especially heavy users having 80% higher chances of hypertension [10]. In addition, Yankey and colleagues concluded, with similar national outpatient data, that increase in marijuana use each year was not only significantly associated with hypertension but with metabolic syndrome as well [11]. In addition, Fong et al. reported a 1.6-fold increase in malignant hypertension among individuals with CUD and a rising trend in the frequency of admissions between 2007 and 2014, especially among young patients (18–44 years) with CUD+ compared to elderly patients [3]. Adrenergic stimulation, alongside parasympathetic nervous system inhibition, causing a positive chronotropic effect, vasoconstriction, and increased blood flow might be the best plausible explanation of hypertension in cannabis users [12,13]. On the other spectrum of HC+ and CUD+ relation, Spindle et al. recounted acute effects of cannabis in healthy infrequent-cannabis users as a transient increase in heart rate and a significant decrease in systolic BP with 10 mg of smoked cannabis [14]. In the elderly (with mean age of 70 years), a reduction in 24 h systolic and diastolic BP has been demonstrated after cannabis use [15]. In another large-population observational study, Haleem et al. showed no association between hypertension and cannabis use [7].

In this study from 2015 to 2017, the propensity matched CUD+ cohort admitted for HC consisted of a higher proportion of black patients (64.8%), and patients with a greater prevalence of smoking, alcohol, and drug addiction vs. CUD- cohort. Consistently, Kennedy et al. from a national survey revealed that African American young adults were more likely than whites to use cannabis before tobacco [16]. In an NHANES survey, non-Hispanic white people (55.7%) had considerably higher hypertension control rates than non-Hispanic black adults (48.5%), non-Hispanic Hispanic (43.5%), and Hispanic (47.4%). Furthermore, concomitant substance abuse may play a vital role in predicting the risk of future cardiovascular events in young adults with CUD+. Prior reports suggested a link between cigarette smoking and earlier onset and increased frequency of cannabis use, as well as a higher incidence of cannabis use disorder symptoms [17]. The combination of both alcohol use disorder and CUD+ was linked to heavier drinking habits and more marijuana difficulties than each substance’s disordered use alone [18].

Our propensity-score matched analysis showed a higher rate of comorbidities such as prior myocardial infarction, chronic pulmonary, liver disease, depression, and psychosis in HC + CUD+ admissions. Cannabis usage was linked to the prevalence of coronary artery disease, after accounting for established cardiovascular disease risk variables, in a national survey (2011–2018) [19]. Recent studies show that CUD+ is associated with depression, especially in young men during adolescence, while the depression is stronger in women during midlife [20]; serotonin may mediate a potential genetic correlation between CUD+ and major depression [21]. Forti et al. conducted a multi-center case–control study and found that daily use of high-potency cannabis can raise the risk of a psychotic disorder by up to five times [22]. Independent association of cannabis use with increased risk of arrhythmias has been reported in young adults with comorbid depression, however, data remains limited to define the long-term effect of depression and cannabis use on systolic or diastolic blood pressure [23]. A study on the elder population reported a greater risk of respiratory symptoms and chronic obstructive pulmonary disease (COPD) when smoking both tobacco and cannabis than tobacco alone [24]. Contrary to this, Gunasekaran et al. reported cannabis users had statistically significantly lower odds of in-hospital mortality in cannabis users than non-cannabis users, among hospitalized COPD patients [25]. Meanwhile, Adejumo et al. showed that cannabis use is associated with decreased incidence of liver disease in alcohol users [26]. Our study revealed lower rates of DM, PVD, hyperlipidemia, obesity, and renal failure in the CUD+ cohort.

Hypertensive crisis significantly increases the long-term risk of other acute cardiac events as established in a 10-year follow-up study by Lee and colleagues [27]. Therefore, it is important to evaluate the risk of major cardiac events in HC patients admitted with known CUD. In the adjusted multivariable analysis, we found significantly higher odds of all-cause mortality by 5.7 folds, the arrhythmia by 1.7 folds, and stroke by 1.4 folds in the CUD+ cohort admitted for HC compared to CUD- cohort. These results are consistent with previous studies showing an association of cannabis use with increased burden and risk of cardiac arrhythmias [28], including atrial fibrillation, atrial flutter, atrioventricular block, premature ventricular contractions, premature atrial contractions, ventricular tachycardia, and ventricular fibrillation that can be life-threatening. Our previous analysis has also confirmed the role of cannabis in higher correlation with arrhythmia and stroke irrespective of concomitant substance abuse [29]. A systematic analysis by Richards et al. highlighted the cannabis-associated increased risk of both acute coronary syndrome and chronic cardiovascular disease [30]. Endocannabinoids are detected in heart tissues and are suggested to be involved in the regulation of heart rate and blood [31]. Though our study did not show statistically significant acute myocardial infarction and cardiac arrest in CUD+, it did show higher odds for these events in CUD+ cohort without reaching a statistical significance. In our previous studies using the National Inpatient Sample and Behavioral Risk Factor Surveillance System (BRFSS) database from the CDC, we observed rising trends and positive associations of cannabis use with stroke events in young adults [32,33]. This is thought to be multifocal angiopathy in young individuals [34] or a cannabis-related consequence of arterial obstruction from a post-myocardial infarction left ventricular thrombus [35]. Though the frequency of tobacco use disorder, alcohol abuse, depression, history of previous myocardial infarction, valvular heart disease and pulmonary circulation disease were higher in HC hospitalizations for CUD+ cohort than CUD-, these comorbidities were not independently associated with higher MACCE in CUD+ compared to CUD-. As a result, according to our population-based study, these characteristics do not appear to operate as effect modifiers for HC-related MACCE for cannabis use status. The risk of the composite endpoint of major adverse cardiac and cerebrovascular events when controlled for confounders trended higher but did not reach a statistical significance (adjusted OR: 1.16, 95% CI: 0.91–1.47, *p* = 0.231).

This study has some limitations that should be considered while interpreting its results. First, there is a possibility of ICD-10 coding error and selection bias as the NIS is an administrative dataset even with the use of validated codes. Additionally, due to the retrospective nature of the dataset, we could not assess the duration, mode, and dose of administration, frequency of cannabis use, or specific cause of death. Moreover, anti-hypertensive medication history of the patients was not available in the NIS. The severity of HC events was not reported. However, this study offers the first perspective into this understudied subject using a large nationwide cohort and invites future prospective studies to further evaluate the association of CUD+ with HC and subsequent short-term risk of MACCE.

## 5. Conclusions

This population-based study showed that the CUD+ cohort had a higher prevalence and higher odds of HC-related admissions in overall young population and young adults with known benign hypertension compared to CUD- cohort. Furthermore, there were significantly higher odds of all-cause mortality, arrhythmia, and stroke in young adults admitted for HC with CUD+ when compared with CUD-. Large population-based prospective studies are warranted to better understand the association of cannabis use with HC and related adverse cardiovascular and cerebrovascular events given the increasing prevalence of cannabis use in the population.

## Figures and Tables

**Table 1:** Baseline Characteristics of Hospitalizations in Young (18–44 years) Patients with versus without Cannabis Use Disorder.

		CUD-	CUD+	Total Young Admissions
		(*n* = 18,824,587)	(*n* = 623,715)	(*n*= 19,448,302)
Age (years) at admission	Median [IQR]	31 (26–37)	29 (24–36)	31 (25–37)
Sex	Male	25.50%	61.20%	26.60%
	Female	74.50%	38.80%	73.40%
Race	White	54.30%	53.10%	54.30%
	African American	18.80%	29.00%	19.10%
	Hispanic	17.80%	12.10%	17.60%
	Asian or Pacific Islander	4.10%	1.20%	4.00%
	Native American	0.80%	1.20%	0.80%
	Others	4.10%	3.40%	4.10%
Primary expected payer	Medicare	6.20%	9.00%	6.30%
	Medicaid	39.20%	49.90%	39.50%
	Private including HMO	43.40%	22.40%	42.70%
	Self-pay/No charge/Others	11.20%	18.70%	11.40%
Median household income national quartile for patient ZIP Code	0–25th	31.80%	40.80%	32.10%
	26–50th	25.40%	25.40%	25.40%
	51–75th	23.50%	20.20%	23.40%
	76–100th	19.20%	13.70%	19.10%
Non-elective admission		68.10%	88.00%	68.70%
Location/teaching status of hospital	Rural	8.20%	8.20%	8.20%
	Urban non-teaching	23.10%	23.40%	23.10%
	Urban teaching	68.70%	68.40%	68.70%
Region of hospital	Northeast	17.40%	21.60%	17.50%
	Midwest	21.30%	24.70%	21.40%
	South	39.60%	35.50%	39.50%
	West	21.70%	18.20%	21.60%
Comorbidities				
Hypertension		19.40%	17.20%	19.40%
Diabetes mellitus		6.50%	5.40%	6.50%
Hyperlipidemia		4.70%	4.40%	4.70%
Obesity		12.20%	7.80%	12.10%
Peripheral vascular disease		0.60%	0.50%	0.60%
Tobacco use disorder		23.10%	58.80%	24.20%
Drug abuse		5.50%	86.20%	8.10%
Alcohol abuse		3.90%	19.10%	4.40%
Acquired immune deficiency syndrome		0.20%	0.60%	0.30%
Rheumatoid arthritis/collagen vascular heart disease		1.20%	0.70%	1.20%
Coagulopathy		3.20%	2.40%	3.20%
Congestive heart failure		1.20%	1.10%	1.20%
Chronic pulmonary disease		8.60%	12.80%	8.70%
Pulmonary circulation disease		0.40%	0.30%	0.40%
Chronic kidney disease		2.70%	2.00%	2.60%
Liver disease		2.40%	3.00%	2.40%
Other neurological disorders		4.10%	6.50%	4.20%
Depression		7.90%	13.60%	8.00%
Psychoses		3.50%	10.00%	3.70%

All *p* < 0.001, CUD = cannabis use disorder, IQR-interquartile range, HMO = health maintenance organization.

**Table 2:** Association Of Cannabis Use Disorder With Hypertensive Crisis-related Hospitalizations In Overall Young Adult Population Additionally, Young Adults With Known Benign Hypertension.

	Overall Young Population			Young Adults with Known Benign Hypertension		
	OR	95%CI	*p*	OR	95%CI	*p*
Unadjusted	1.52	1.41–1.64	<0.001	1.25	1.09–1.44	0.002
Model A: Adjusted with sociodemographic and hospital characteristics	0.97	0.89–1.04	0.394	1.12	0.97–1.30	0.123
Model B: Model A + comorbidities including alcohol abuse and tobacco use disorder	1.22	1.13–1.32	<0.001	1.17	1.01–1.36	0.034
Model C: Model B + cocaine abuse and other stimulant abuse including amphetamine	1.15	1.06–1.24	0.001	1.12	0.96–1.30	0.154

*p* < 0.05 indicates statistical significance. OR = odds ratio, CI = confidence interval. Sociodemographic and hospital characteristics included-age at admission, sex, race, elective versus non-elective admission, primary expected payer, median household income national quartile for patient zip code, bed size of hospital, location/teaching status of hospital, region of hospital. Comorbidities included- deficiency anemias, acquired immune deficiency syndrome, rheumatoid arthritis/collagen vascular diseases, coagulopathy, congestive heart failure, valvular heart disease, peripheral vascular disease, depression, other neurological disorders, chronic pulmonary disease, diabetes mellitus, hyperlipidemia, obesity, renal failure, fluid and electrolyte disorders, liver disease, hypothyroidism, solid tumor without metastasis, metastatic cancer, lymphoma, prior mi, and prior transient ischemic attack/stroke along with substance abuse as indicated in models built for multivariable regression analyses.

**Table 3:** Baseline characteristics of Propensity-score Matched Cohorts (CUD+ versus CUD-) With Hypertensive Crisis-related Hospitalizations.

		CUD- (*n* = 4440)	CUD+ (*n* = 4440)	Total HTN Crisis in Young (*n* = 8880)	*p*
Age (years) at admission	Median [IQR]	37 (31–41)	36 (31–40)	36 (31–40)	0.004
Sex	Male	62.80%	64.20%	63.50%	0.186
	Female	37.20%	35.80%	36.50%	
Race	White	18.90%	20.80%	19.90%	0.029
	African American	65.10%	64.40%	64.80%	
	Hispanic	11.80%	10.20%	11.00%	
	Asian or Pacific Islander	0.70%	0.80%	0.70%	
	Native American	1.10%	0.90%	1.00%	
	Others	2.40%	2.80%	2.60%	
Primary expected payer	Medicare	15.10%	15.50%	15.30%	0.403
	Medicaid	48.20%	46.70%	47.50%	
	Private including HMO	13.00%	13.00%	13.00%	
	Self-pay	18.50%	19.90%	19.20%	
	No charges	1.70%	1.70%	1.70%	
	Others	3.60%	3.20%	3.40%	
Median household income national quartile for patient ZIP Code	0–25th	63.00%	60.50%	61.70%	0.061
	26–50th	20.40%	21.70%	21.10%	
	51–75th	12.50%	12.80%	12.70%	
	76–100th	4.20%	5.00%	4.60%	
Elective versus non-elective admission	Non-elective	97.70%	97.60%	97.70%	0.724
	Elective	2.30%	2.40%	2.30%	
Bed size of hospital	Small	18.80%	20.40%	19.60%	0.145
	Medium	30.00%	28.80%	29.40%	
	Large	51.20%	50.80%	51.00%	
Location/teaching status of hospital	Rural	6.90%	5.50%	6.20%	0.017
	Urban non-teaching	16.80%	17.90%	17.30%	
	Urban teaching	76.40%	76.60%	76.50%	
Region of hospital	Northeast	14.60%	13.00%	13.80%	0.058
	Midwest	20.30%	21.60%	20.90%	
	South	49.40%	49.00%	49.20%	
	West	15.70%	16.40%	16.00%	
Comorbidity					
Diabetes mellitus		31.30%	24.00%	27.60%	<0.001
Hyperlipidemia		19.40%	18.50%	18.90%	0.278
Obesity		28.00%	24.10%	26.10%	<0.001
Peripheral vascular disease		2.30%	2.10%	2.20%	0.717
Tobacco use disorder		40.80%	65.40%	53.10%	<0.001
Drug abuse		8.40%	87.70%	48.10%	<0.001
Alcohol abuse		5.60%	12.80%	9.20%	<0.001
Acquired immune deficiency syndrome		1.20%	0.60%	0.90%	0.001
Rheumatoid arthritis/collagen vascular disease		2.90%	2.70%	2.80%	0.521
Coagulopathy		5.70%	3.40%	4.60%	<0.001
Congestive heart failure		17.30%	16.20%	16.80%	0.156
Valvular disease		1.90%	3.80%	2.90%	<0.001
Chronic pulmonary disease		12.50%	14.20%	13.30%	0.019
Pulmonary circulation disease		0.20%	0.80%	0.50%	<0.001
Renal failure		45.30%	34.30%	39.80%	<0.001
Liver disease		2.50%	4.30%	3.40%	<0.001
Other neurological disorders		7.30%	7.80%	7.50%	0.422
Depression		8.30%	11.00%	9.70%	<0.001
Psychoses		4.40%	7.80%	6.10%	<0.001
Prior Myocardial infarction		3.30%	4.30%	3.80%	0.012
Prior Stroke/Transient ischemic attack		4.40%	4.40%	4.40%	1

*p* < 0.05 indicates statistical significance. CUD = cannabis use disorder, IQR-interquartile range, HTN = hypertensive, HMO = health maintenance organization.

**Table 4:** In-hospital Outcomes of Hospitalizations for Hypertensive Crisis in Young Adults with vs. without Cannabis Use Disorder.

		CUD- (*n* = 4440)		CUD+ (*n* = 4440)		Total HTN CRISIS IN YOUNG (*n* = 8880)		*p*	Adjusted Odds ratio	95% CI		Adjusted *p*
Composite major adverse cardiac/cerebrovascular events, MACCE		475	10.7%	490	11.0%	965	10.9%	0.609	1.16	0.91	1.47	0.231
All-cause mortality		25	0.6%	35	0.8%	60	0.7%	0.192	5.74	2.55	12.91	<0.001
Acute myocardial infarction		215	4.8%	270	6.1%	485	5.5%	0.01	1.26	0.91	1.73	0.166
Arrhythmia		430	9.7%	500	11.3%	930	10.5%	0.015	1.73	1.38	2.17	<0.001
Stroke		240	5.4%	215	4.8%	455	5.1%	0.229	1.46	1.02	2.10	0.040
Cardiac arrest including ventricular fibrillation/flutter		25	0.6%	20	0.5%	45	0.5%	0.455	2.75	0.99	7.66	0.053
Disposition of patient	Routine	3575	80.5%	3485	78.7%	7060	79.6%	<0.001				
	Transfer to short term hospital	110	2.5%	70	1.6%	180	2.0%					
	Other transfer including SNF, ICF, etc.	150	3.4%	135	3.0%	285	3.2%					
	Home health care	185	4.2%	195	4.4%	380	4.3%					
Length of stay (days), median [IQR]		3 (2–5)		3 (2–5)		3 (2–5)		0.223				
Cost adjusted for inflation in 2017 (USD), median [IQR]		7074 (4429–11519)		6948 (4768–12,063)		6999 (4647–11,746)		0.187				

*p* < 0.05 indicates statistical significance. CUD = Cannabis use disorder, MACCE= Major adverse cardiac and cerebrovascular events, SNF = skilled nursing facility, ICF = intermediate care facility. Multivariable regression analysis was adjusted for age, sex, race, type of admission(elective/non-elective), median household income quartile of patients’ zip code, payer status, hospital bed size, location and teaching status, and comorbidities including diabetes, hyperlipidemia, obesity, peripheral vascular disease, overall substance abuse, alcohol abuse, tobacco use disorder, chronic kidney disease, coagulopathy, congestive heart failure, valvular heart disease, chronic obstructive pulmonary disease, pulmonary circulation disorder, depression, other neurological disorders, fluid-electrolyte disorders, and prior history of myocardial infarction or transient ischemic attack/stroke.

**Table 5:** Predictors of MACCE in Hypertensive Crisis-related Admissions among Young Patients with Cannabis Use Disorder.

	aOR	95% CI		*p*
		Lower	Upper	
Age (years) at admission	1.04	1.02	1.06	<0.001
Male vs. Female	1.19	0.95	1.5	0.136
Race				0.001
African American vs. white	0.67	0.52	0.86	0.002
Hispanic vs. white	0.8	0.54	1.18	0.254
Others vs. white	1.85	1.08	3.18	0.025
Primary expected payer				<0.001
Medicaid vs. Medicare	1.56	1.11	2.18	0.011
Private including HMO vs. Medicare	1.61	1.07	2.43	0.023
Median household income national quartile for patient ZIP Code				<0.001
26–50th vs. 0–25th	0.61	0.47	0.81	<0.001
51–75th vs. 0–25th	1.41	1.06	1.87	0.019
76–100th vs. 0–25th	0.16	0.06	0.41	<0.001
Region of hospital				<0.001
Midwest vs. Northeast	2.06	1.37	3.09	0.001
South vs. Northeast	1.95	1.32	2.89	0.001
West vs. Northeast	1.06	0.66	1.71	0.801
Comorbidities				
Acquired immune deficiency syndrome	5.42	2.24	13.1	<0.001
Peripheral vascular disease	3.03	1.79	5.11	<0.001
Coagulopathy	2.07	1.33	3.22	0.001
Prior myocardial infarction	1.57	0.96	2.56	0.07
Prior TIA/Stroke	1.57	1.02	2.4	0.039
Hypothyroidism	1.5	0.79	2.85	0.213
Other neurological disorders	1.5	1.03	2.18	0.035
Pulmonary circulation disease	1.39	0.5	3.88	0.53
Obesity	1.25	1	1.58	0.053
Tobacco use disorder	1.23	0.98	1.54	0.076
Hyperlipidemia	1.2	0.93	1.55	0.168
Valvular heart disease	1.05	0.62	1.8	0.852
Alcohol abuse	1.01	0.75	1.37	0.941
Chronic kidney disease	0.94	0.74	1.2	0.61
Diabetes mellitus	0.89	0.7	1.15	0.374
Depression	0.72	0.49	1.05	0.086
Congestive heart failure	0.67	0.49	0.92	0.014
Chronic pulmonary disease	0.44	0.31	0.62	<0.001

*p* < 0.05 indicates statistical significance, aOR = adjusted odds ratio, CI = confidence interval, HMO-health maintenance organization, TIA = transient ischemic attack. MACCE= composite major adverse cardiac and cerebrovascular events were defined as all-cause inpatient mortality, acute myocardial infarction, arrhythmia, cardiac arrest including ventricular fibrillation/flutter, and stroke.

## Data Availability

We used a publically available anonymous national database, i.e., National Inpatient Sample (datasets from October 2015 to December 2017).
